# OCT-angiography: A qualitative and quantitative comparison of 4 OCT-A devices

**DOI:** 10.1371/journal.pone.0177059

**Published:** 2017-05-10

**Authors:** Marion R. Munk, Helena Giannakaki-Zimmermann, Lieselotte Berger, Wolfgang Huf, Andreas Ebneter, Sebastian Wolf, Martin S. Zinkernagel

**Affiliations:** 1 Department of Ophthalmology, Inselspital, Bern University Hospital, University of Bern, Switzerland; 2 Bern Photographic Reading Center, University of Bern, Switzerland; 3 Department of Clinical Research, Inselspital Bern University Hospital, University of Bern, Switzerland; 4 Center for Medical Physics and Biomedical Engineering, Medical University of Vienna, Vienna, Austria; Justus Liebig Universitat Giessen, GERMANY

## Abstract

**Purpose:**

To compare the quality of four OCT-angiography(OCT-A) modules.

**Method:**

The retina of nineteen healthy volunteers were scanned with four OCT-devices (Topcon DRI-OCT Triton Swept-source OCT, Optovue RTVue-XR, a prototype Spectralis OCT2, Heidelberg-Engineering and Zeiss Cirrus 5000-HD-OCT). The device-software generated en-face OCT-A images of the superficial (SCP) and deep capillary plexuses (DCP) were evaluated and scored by 3 independent retinal imaging experts. The SCP vessel density was assessed using Angiotool-software. After the inter-grader reliability assessment, a consensus grading was performed and the modules were ranked based on their scoring.

**Results:**

There was no significant difference in the vessel density among the modules (Zeiss 48.7±4%, Optovue 47.9±3%, Topcon 48.3±2%, Heidelberg 46.5±4%, p = 0.2). The numbers of discernible vessel-bifurcations differed significantly on each module (Zeiss 2±0.9 bifurcations, Optovue 2.5±1.2, Topcon 1.3±0.7 and Heidelberg 0.5±0.6, p≤0.001). The ranking of each module differed depending on the evaluated parameter. In the overall ranking, the Zeiss module was superior and in 90% better than the median (Bonferroni corrected p-value = 0.04). Optovue was better than the median in 60%, Topcon in 40% and Heidelberg module in 10%, however these differences were not statistically significant.

**Conclusion:**

Each of the four evaluated OCT-A modules had particular strengths, which differentiated it from their competitors.

## Introduction

OCT-angiography (OCT-A) is a new diagnostic tool and heavily promoted as an alternative or an adjunct to classic fluorescein angiography (FA). It is a fast imaging tool, detecting streaming blood, thereby allowing to construct an image of the retinal vasculature; in contrast to “classical” FA it is dye-free, and therefore lacks significant side effects associated with the fluorescein injections such as such as vomiting, hypersensitivity reactions and cardiovascular complications. [1] This new technology allows the in situ, high-resolution visualization of the individual vascular layers. In contrast to FA, which displays only the superficial capillary network, OCT-A visualizes the superficial, the deep and the choroidal vascular network; even the middle capillary plexus can be identified. [2].

Several OCT manufacturers now offer OCT devices including algorithms enabling the practitioner to obtain regular OCT B-scans as well as volumetric angiographic images. Different techniques such as Doppler shift, speckle variance/decorrelation, phase variance, optical micro-angiography and correlation mapping are employed to differentiate blood vessels by depicting the change in the OCT-signal induced by the moving blood cells. [3,4] So far Angiovue optical coherence tomography angiography (Optovue RTVue XR Avanti, Optovue Inc., Fremont, CA) based on a split spectrum amplitude decorrelation angiography algorithm (SSADA), Zeiss AngioPlex (Cirrus HD-OCT 5000, Zeiss Meditec. Inc.) based on micro-angiography (OMAG) and SS-OCT Angiography employed in a Swept source OCT DRI OCT Triton (Topcon DRI OCT Triton Swept source OCT, Topcon, Japan) using the so called OCT angiography ratio analyses (OCTARA) algorithm are commercially available. Prototypes of the Spectralis OCT2 module (Heidelberg Engineering, Germany) with a full spectrum amplitude decorrelation algorithm, and a prototype of AngioScan (RS-3000 Advance OCT, Nidek Co., Ltd., Japan) based on a complex décor relation algorithm were introduced and are currently tested. Further there are other OCT-A modules under development such as the OCT-A system inbuilt in the Copernicus Revo and REVO NX by OPTOPOL.

Previous studies aimed to compare the performance of different OCT-A techniques applied in the above listed OCT-A modules including phase variance, absolute complex difference, speckle variance and absolute intensity difference. It was confirmed that all methods generate excellent flow motion contrast images, with phase variance and absolute complex difference methods requiring more complex analyses than intensity based algorithms such as speckle variance and absolute intensity difference. [5] A recent study by De Vitis et al. compared the AngioVue (Optovue) with the Angioplex (Zeiss) and found that the Angioplex required shorter acquisition time and showed a lower number of motion artefacts when compared to the Angiovue.{De Vitis, 2016 #881}.

However so far no data are available which systematically compare the commercially available OCT-A modules.

The aim of this study therefore was to compare the quality of 4 different commercially available OCT-A modules in healthy subjects.

## Methods

### Patients and setting

Nineteen healthy subjects were evaluated in this cross sectional study. Subjects had a visual acuity of 20/20 or better without a clinical history and without any evidence of an eye disease including retinal disease or glaucoma. Exclusion criteria were also the presence of diabetes or hypertension or any other cardiovascular disease. The retina was scanned using a Zeiss Cirrus 5000 HD-OCT (Zeiss Meditec. Inc, Germany), an Angiovue, RTVue XR Avanti (Optovue, Inc), a Topcon DRI OCT Triton Swept source OCT and a prototype of Spectralis OCT2 (Heidelberg Engineering, Germany). The Topcon DRI Swept source (SS)- OCT used a wavelength of 1050nm, whereas the remaining devices used shorter wavelengths around 800nm (870nm, Heidelberg; 840nm, Optovue and Zeiss). The Zeiss Cirrus HD-OCT Model 5000 with Angioplex uses a so called OCT- microangiography complex algorithm (OMAG) and an A- scan rate of 68Khz. OMAG identifies changes in the phase and intensity information of the OCT scans to quantify motion contrast.[6] For eye tracking the FastTrac technology is implemented and the retina is sampled 15 frames per second to minimize motion artefacts. Only areas which may be affected by motion artefacts are rescanned, which decreases the acquisition time. A 3x3 pattern with a 245x245 resolution was chosen, with a mean distance of 12.2 microns between each scan and each B-scan was repeated 4 times at the same position. The A scan depth is 2mm with an axial resolution of 5 μm and a transverse resolution of 15 μm.[6] The Optovue Angiovue utilizes SSADA, which splits the spectrum into different, small bands while employing a decorrelation measure. [7] With Optovue, a 3x 3-volume scan centered on the fovea was obtained with an A-scan rate of 70kHz. Each volume scan consists of 304x304 A-scans with 2 consecutive B-scans at each position. Two right angled OCT-A volumes scans are performed for orthogonal registration to correct for motion artefacts. [7] OCTA Ratio Analyses (OCTARA) employed by Topcon is an intensity ratio analyses and is not based on amplitude decorrelation. It does not require splitting the spectrum and therefore preserve axial resolution, which is important as SS-OCT achieve a somewhat lower axial resolution.[8] The SS-Topcon device has a 100KHz A scan rate using a wavelength of 1050nm. A 3x3 volume scan was performed and each B-scans position was automatically scanned four times.[8] The Heidelberg prototype uses a full-spectrum amplitude decorrelation algorithm (FS-ADA) to evaluate motion contrast, which allows the evaluation of blood flow without sacrificing depth resolution. [9] The prototype acquired an A-scan rate of 85kHz with an axial resolution of 7μm and a lateral resolution of 14μm.[9] The available volume scan pattern was 4.3x 1.5 mm with 11μm between each B-scan. The ART frame was set at 13 frames per scan. Truetrack was employed to control for eye movements and minimize motion artefacts.

**Fig 1:**
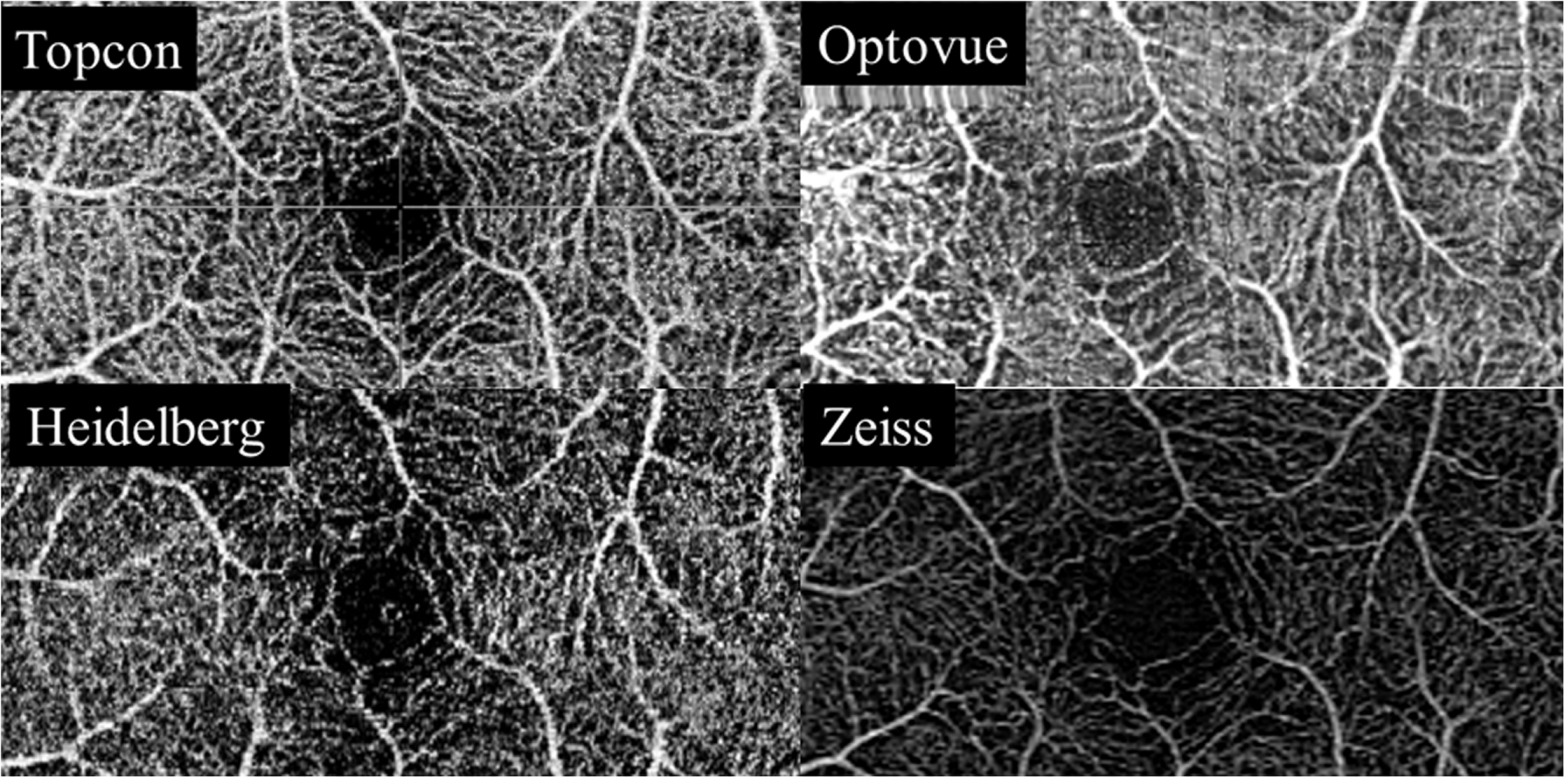
Superficial capillary plexus. Representative en face scans of the superficial capillary plexus (SCP) using the Swept source OCT Angio Topcon DRI OCT Triton (Top left), the Angiovue Optovue RTVue XR Avanti, (Top right), the Prototype of Spectralis OCT2 module with full spectrum decorrelation algorithm, Heidelberg Engineering (Bottom left) and the Zeiss AngioPlex Cirrus 5000 HD-OCT (Bottom right).

**Fig 2:**
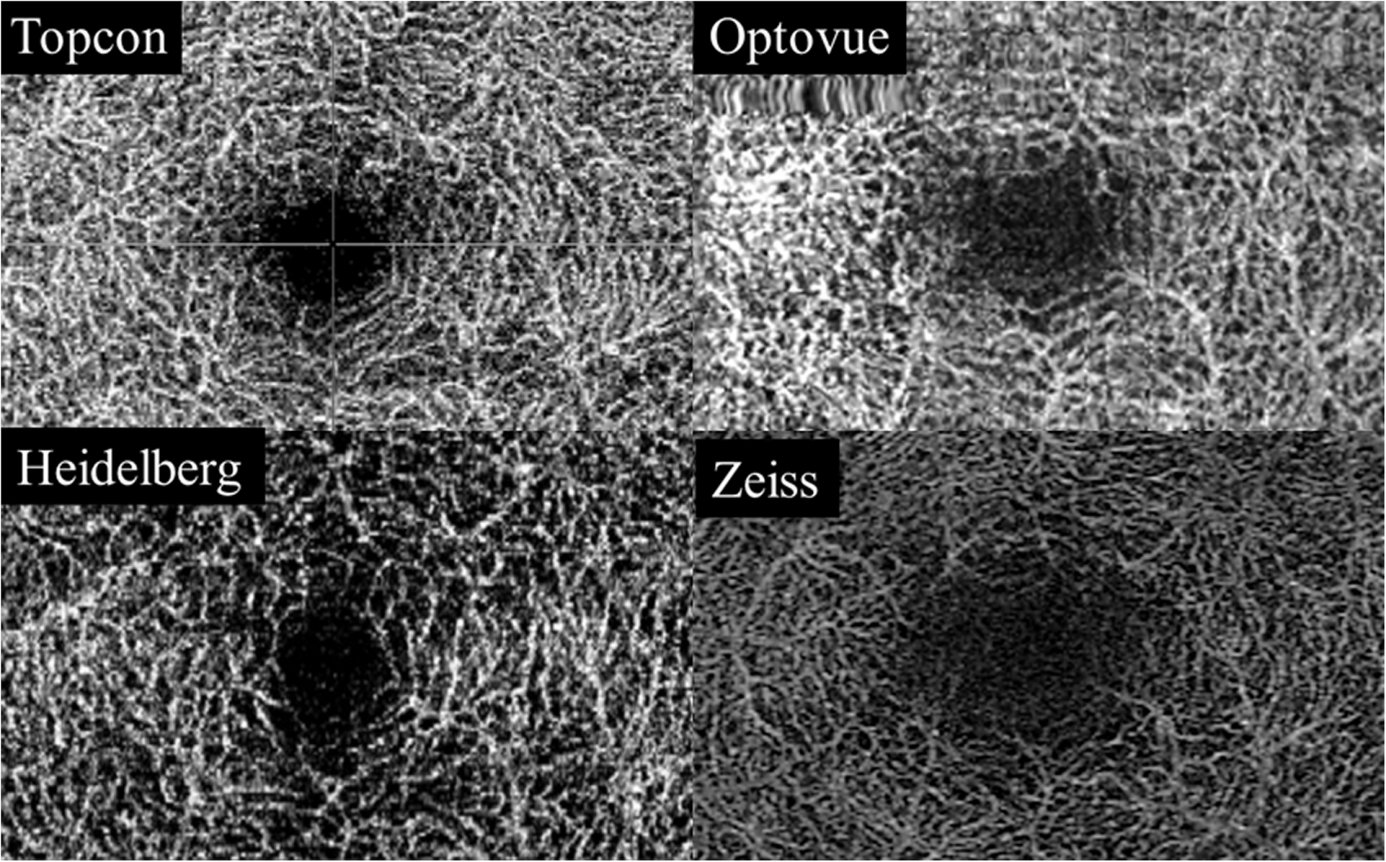
Deep capillary plexus. Representative en face scans of the deep capillary plexus (SCP) using the Swept source OCT Angio Topcon DRI OCT Triton (Top left), the Angiovue Optovue RTVue XR Avanti, (Top right), the Prototype of Spectralis OCT2 module with full spectrum decorrelation algorithm, Heidelberg Engineering (Bottom left) and the Zeiss AngioPlex Cirrus 5000 HD-OCT (Bottom right).

The study adhered to the tenets of the Declaration of Helsinki and was approved by the local ethics committee at Inselspital. A weaver of informed consent was granted due to the use of anonymized data (KEK number 2016–02100). The superficial (SCP) as well as deep vascular plexus (DCP) were segmented using the inbuilt software on each device. The location of the segmentation line of the SCP and the DCP of each module can be found in S1 Table in S1 Table and S2 Table file. Representative images for each module for the SCP and DCP are shown in Figs 1 and 2. The scans were checked for segmentation errors and subsequently the four separate, device-software generated en-face OCT-A images of the SCP and DCP from each volunteer were exported, analyzed and scored by 3 independent, experienced retinal imaging experts according to a pre-specified grading protocol.

### Grading protocol

The grading protocol included qualitative as well as quantitative parameters: Motion artefacts (1 = no artefacts, 0 = some artefacts, -1 = severe motion artefacts), image artefacts (1 = no artefacts, 0 = some artefacts, -1 = severe image artefacts), the distinguishability of the foveal avascular zone (FAZ) (1 = FAZ border good distinguishable, 0 = middle, -1 = FAZ border barely/not distinguishable), and the vessel continuity and discriminability of large and small vessels (1 = vessel continuity clearly preserved, 0 = vessel continuity partly preserved, -1 = vessel continuity not preserved) were assessed. The number of clearly identifiable bifurcations identifiable on the superficial en-face image was counted on each scan. Therefore a main, large vessel branch at 12 o`clock was chosen and the number of identifiable, subsequent bifurcations towards the terminal capillary end were counted on the respective branch. Additionally, the superficial layer retinal vessel density was assessed using the publicly available software Angiotool. [10] Vessel density was defined as the area occupied by vessel lumens following binary reconstruction of images. [11].

Motion artifacts (or displacement artefacts) were considered as present when there were characteristic white-line artefacts present on OCT-A image with corresponding discontinuity at the en-face image in the B-scan direction or lateral displacement of parts of the image or doubling of retinal vessels. Axial motion artefacts resulting from breathing, tremor or pulsations as well as transverse motion artefacts caused by loss/change and saccades were evaluated.[12,13] Blink lines, identifiable as continuous dark lines of varying width visible on each singular slab were also included in this category. [14] Image artefacts were defined as any anomaly in the visual representation of information of the SCP and DCP slabs derived from the scanned object aside from motion artefacts. [12,14] These artefacts included segmentation artefacts, leading to deviation of the slab and projection artifacts, which were assumed when there were vessels seen clearly at deeper location than they actually inhabit.[12,15,16] It further included “negative projections” derived from superincumbent vessels after removing the projection flow signal using available device inbuilt software.

**Fig 3:**
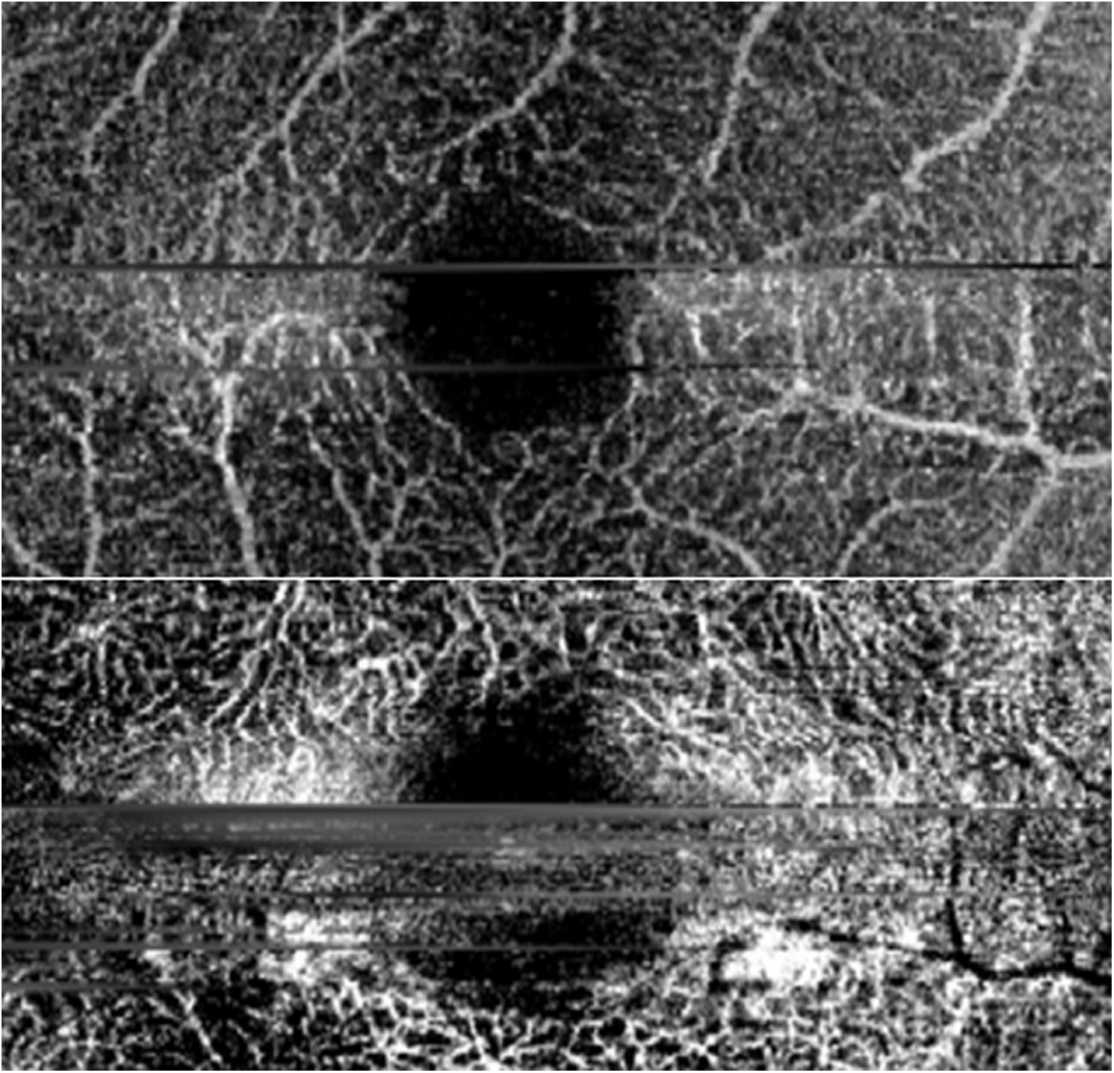
Severe motion artefacts. Representative examples of severe motion artefacts.

**Fig 4:**
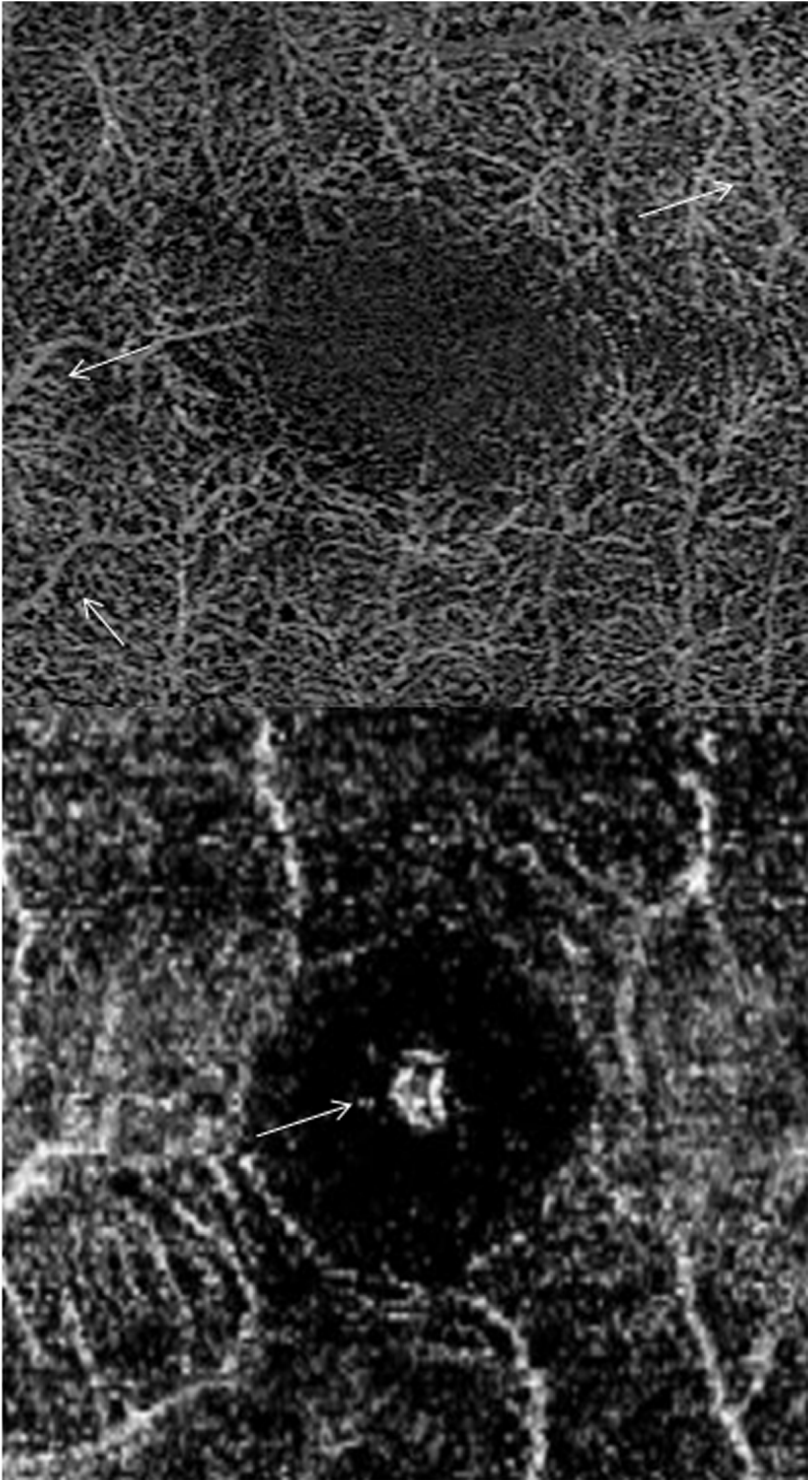
Projection artefacts and further examples of evaluated parameters. Top: Representative example of some projection artefacts (white arrows). Bottom: Example of “vessel continuity not preserved” of the small and large vessels. Central specular dots (white arrow) a form of image artefact, which can be seen in healthy eyes is shown.

The parameter “some artefact” was defined as the presence of at least one artefact of respective category. The presence of 5 or more artefacts of respective category was considered as severe artefacts. Artefacts making reasonable evaluation of the microvasculature impossible were considered as severe artefacts as well. This included also broad artefacts as large as 5% of the image width or length in horizontal and vertical directions, respectively. Some representative examples of artifacts can be found in Figs 3 and 4.

After concordance analyses and the evaluation of the inter-grader reliability, a consensus grading was performed. The consensus dataset was then used to compare, score and rank the four modules.

### Statistical analyses

Statistical analyses were performed with SPSS (IBM, SPSS statistics, Version 21, SPSS Inc., Chicago) software and R (www.r-project.org). Fleiss kappa coefficient was employed to quantify inter-grader reliability. After the evaluation of the interrater reliability, a consensus grading was performed and a final score ranging from -1/0/+1 was given for each feature for each image. These scores were then summed up and normalized with a maximal and minimal scoring ranging from -100 to +100. Based on the scores the devices were ranked for each evaluated parameter. Differences in ordinal variables such as motion artefacts, FAZ and vessel continuity were analyzed using Chi-squared test and numeric data such as number of counted bifurcations as well as vessel density were evaluated using ANOVA. An overall ranking of the modules was performed using exact binominal testing. Hereby it was evaluated in how many cases the module was better than the median with the set of evaluated parameters serving as the test sample for all features.

P-values <0.05 were considered statistically significant and Bonferroni Holmes correction was used to adjust for multiple testing. Values are given as mean±SD.

## Results

This study included 19 eyes of 19 healthy volunteers (mean age 35.3±8.2 years). Inter-grader reliability in respect to each OCT-A module can be found in Table 1.

**Table 1:** Inter-grader reliability in respect to individual devices.

	Optovue		Topcon		Zeiss		Heidelberg	
	kappa	p-value	kappa	p-value	kappa	p-value	kappa	p-value
**Motion artefacts SCP**	0.818	<0.0001	0.566	<0.0001	0.527	<0.0001	*0*.*31*	*≤0*.*002*
**Motion artefacts DCP**	0.727	<0.0001	0.6	<0.0001	0.686	<0.0001	*0*.*425*	*<0*.*0001*
**Image artefacts SCP**	0.662	<0.0001	0.1	0.47	0.912	<0.0001	0.165	0.087
**Image artefacts DCP**	0.103	0.259	*0*.*442*	*<0*.*0001*	0.524	<0.0001	0.224	0.022
**FAZ superior**	0.515	<0.0001	*0*.*396*	*<0*.*0001*	0.782	<0.0001	*0*.*31*	*0*.*00122*
**FAZ deep**	0.535	<0.0001	0.107	0.367	*0*.*493*	*<0*.*0001*	*0*.*357*	*0*.*002*
**Large vessel cont SCP**	0.718	<0.0001	*0*.*412*	*0*.*0002*	0.912	<0.0001	0.285	0.007
**Small vessel cont SCP**	0.685	<0.0001	0.644	<0.0001	0.592	<0.0001	*0*.*458*	*<0*.*0001*
**Small vessel cont DCP**	0.613	<0.0001	0.245	0.0132	0.546	<0.0001	0.605	<0.0001
**N of bifurcation**	0.148	0.0453	0.295	0.0003	0.541	<0.0001	*0*.*345*	*0*.*0006*
**Overall reliability SCP**	0.522	<0.0001	0.296	0.0087	0.703	<0.0001	0.208	0.116
**Overall reliability DCP**	0.605	<0.0001	0.584	<0.0001	0.743	<0.0001	0.268	0.0133

Kappa values given in dark grey indicate a strong agreement (kappa value ranging from 0.9–0.7). Kappa values indicating a moderate agreement (= kappa value ranging from 0.5–0.7) are given in light grey. Kappa values of weak agreement (= kappa value ranging from 0.3–0.5) are shown in italic print style and minimal agreements (kappa value ranging from 0.1–0.3) are given in normal print style. Overall reliability indicate the intergrader reliability of all evaluated features in the SCP and the DCP together. Kappa = Fleiss kappa, SCP = superficial capillary plexus, DCP = deep capillary plexus, FAZ = foveal avascular zone, cont. = continuity, N = number.

### OCT-A module differences

There was no difference in the overall vessel density among the evaluated modules using Angiotool (Zeiss 48.7±4%, Optovue 47.9±3%, Topcon 48.3±2%, Heidelberg 46.5±4%, p = 0.2). However although there was no difference in vessel density, the correlation coefficients were rather weak for respective parameter among the modules (Spearman correlation coefficient ranging from r = -0.16–0.35, details see S2 Table in S1 and S2 Table file) No significant difference among the devices in terms of motion artefacts were detected (Table 2). However, for image artefacts of the SCP the Zeiss and the Topcon modules were superior compared to the other two devices (Table 2). The FAZ border of the SCP slabs were best appreciable on the Zeiss images, followed by the Optovue device, whereas the FAZ of the DCP was best discernable on the Optovue device followed by the Heidelberg module. The illustration of the continuity of the vessels was found to be superior on the Zeiss module (Table 2). The ranking of the modules according to each individual evaluated feature can be found in Table 2. The underlying normalized scores can also be found in Table 2.

**Table 2:** Ranking and underlying normalized scores of each module for each evaluated variable of the consensus dataset.

	Ranking (Scores)				p-value
	Optovue	Topcon	Zeiss	Heidelberg	
**Motion artefacts SCP**	**2** (42)	**3** (26)	**1** (74)	**4**(11)	0.135
**Motion artefacts DCP**	**2** (42)	**3** (21)	**1** (63)	**4** (15)	0.076
**Image artefacts SCP**	**3** (58)	**2** (74)	**1** (74)	**4** (-21)	**≤0.001**
**Image artefacts DCP**	**4** (12)	**1** (30)	**2** (19)	**3** (14)	0.32
**FAZ SCP**	**2** (47)	**3** (36)	**1** (84)	**4** (5)	**≤0.001**
**FAZ DCP**	**1** (31)	**3** (-37)	**4** (-58)	**2** (-21)	**0.002**
**Large vessel cont SCP**	**3** (47)	**2** (63)	**1** (74)	**4** (26)	**0.027**
**Small vessel cont SCP**	**2** (53)	**3** (16)	**1** (74)	**4** (-58)	**≤0.001**
**Small vessel cont DCP**	**3** (36.7)	**2** (37.1)	**1** (42)	**4** (-11)	0.071
**N of bifurcation**	**1** (2.5±1.2)	**3** (1.3±0.7)	**2** (2±0.9)	**4** (0.5±0.6)	**≤0.001**

Each image was graded by the readers and was given a score ranging from -1/0/+1. (e.g. Motion artefacts (1 = no artefacts, 0 = some artefacts, -1 = severe motion artefacts). Thereafter a consensus grading was performed and a final score was given for each feature for each image. These scores were then summed up and normalized. The maximal and minimal possible scoring after normalization was -100 to +100 (see numbers in brackets). Based on these scores each device was ranked (bold numbers). Rank of 1 describes highest scores and best quality (in grey). The variable “n of Bifurcation” shows mean and standard deviation of counted bifurcations (see numbers in brackets). Therefore the main, large vessel branch at 12 o`clock was chosen and the number of identifiable, subsequent bifurcations towards the terminal capillary end were counted on the respective branch and respective variable was then ranked based on the number of identifiable bifurcations (bold numbers). Differences among the modules (**p-values**) were calculated using Chi-squared testing.

**Fig 5:**
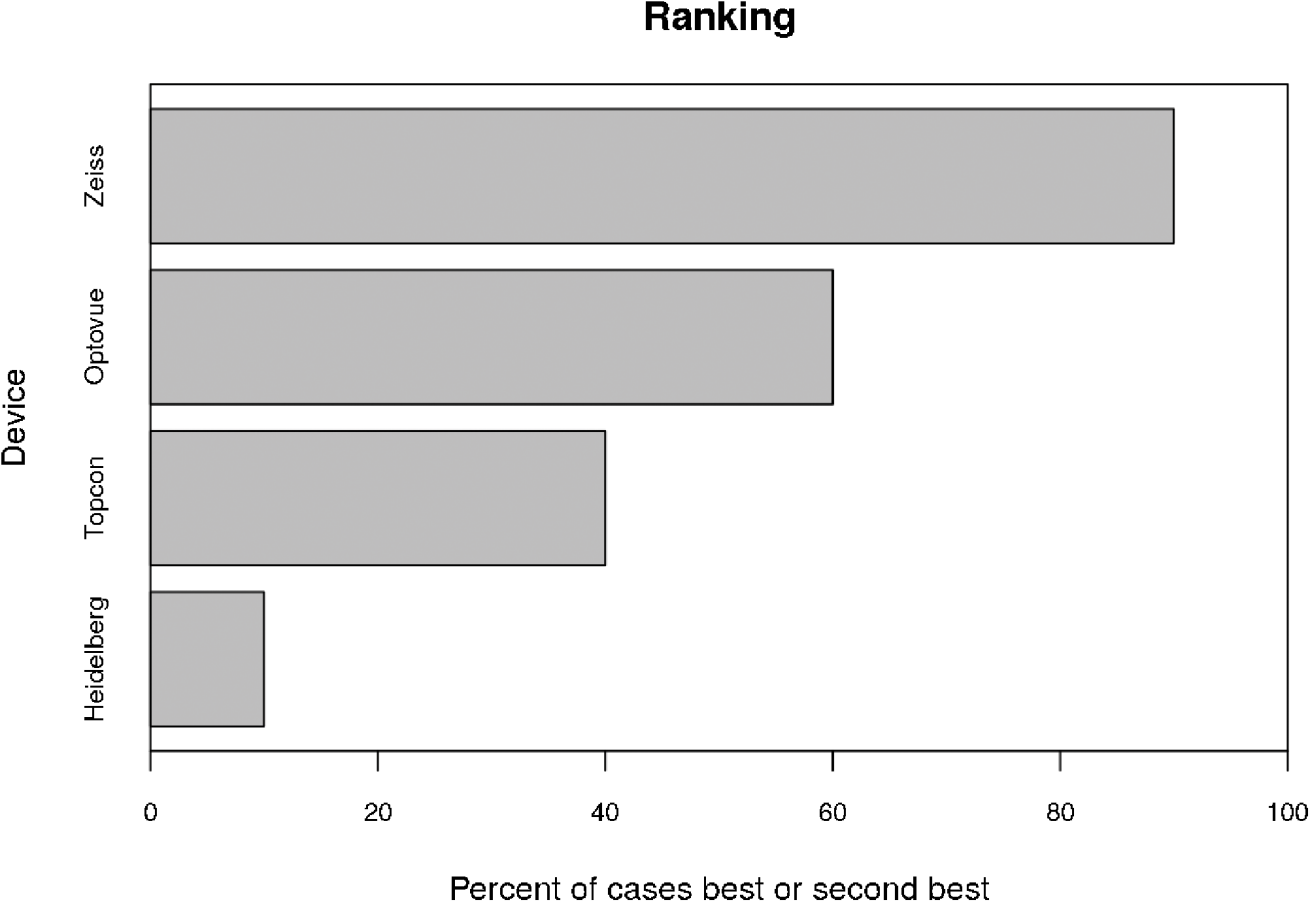
OCT-A module ranking.

Significant different numbers of bifurcations were discernible on each module (Zeiss 2±0.9 bifurcations, Optovue 2.5±1.2, Topcon 1.3±0.7 and Heidelberg 0.5±0.6, p≤0.001, Table 2). In the overall ranking, the Zeiss module was superior and in 90% better than the median (Bonferroni corrected p-value = 0.04, Fig 5). The Optovue was found to be better than the median in 60%, the Topcon in 40% and the Heidelberg module in 10%, however these evaluations missed statistical significance (Fig 5).

Overall ranking of each OCT-A module using exact binominal testing based on the scores of each evaluated feature. In 90% the Zeiss Angioplex module was better than the median (corrected p-value 0.04), The Optovue was in 60%, the Topcon in 30% and the Heidelberg in 10% better than the median. However, differences are not statistically significant.

## Discussion

We here present the first comparison of 4 OCT-A modules. Each of the four modules had particular strengths, which differentiated it from their competitors. Overall, however, the Zeiss module seemed to be the most reliable, accurate and precise device in terms of our evaluated variables, followed by the Optovue, the Topcon and the Heidelberg module. Each module employed different technology to quantify the motion contrast and each module had different approaches to minimize motion artefacts and achieve optimal image quality with high resolution. [6–8,17].

Inter-grader agreement also differed in respect to evaluated feature and device. It seems noteworthy that the reliability of the grading was found to be higher in the Zeiss and Optovue module compared to the Topcon and Heidelberg module. This may be explained by the fact that the grader reliability seems associated with the quality of the images and evaluated features. Images of higher quality depicting the evaluated feature more accurately will result in a stronger inter-grader agreement than poor images. Taking into account that overall the Zeiss module was ranked best and the Optovue device second best, the differences in the inter-grader reliability seem reasonable.

Motion artefacts in the SCP and the DCP were less prominent with the Zeiss Angioplex module, followed by the OCT-A modules of Optovue, Topcon and Heidelberg. However, these findings were not statistically significant. A previous study compared the AngioVue (Optovue) with the Angioplex (Zeiss) and found that the Angioplex required shorter acquisition time and showed a lower number of motion artefacts when compared to the Angiovue.[13] Further the number of low signal strength images and the images impossible to analyze were lower in the Angioplex module compared to the Angiovue module.[13].

In order to prevent motion artefacts manufacturers use different approaches. Zeiss (FastTrack), Heidelberg (TrueTrack) and Topcon (SMARTTRACK) implemented a retinal eye tracking, while the here evaluated Optovue device, used a software based method in which a retinal area is repeatedly scanned horizontally and vertically. This software based approach may account for longer acquisition times compared to the eye tracking as shown in the previous study by De Vities et al. [13] In our study, however it did not seem to impact the severity of motion artefacts as the Optovue module was ranged second best for this evaluated feature. Further, the now available Angiovue modules provide real time eye tracking as well. It has been claimed that the SSADA algorithm mainly accounts for axial artefacts and therefore transverse artefacts may still remain an issue. This assumption was also not confirmed in our analyses. Although not statistically significant, the Zeiss module scored highest in terms of the absence of motion artefacts. This may be explained by the fact that beside the Fasttrack technology for continuous eye tracking, this module also samples the retina 15 times per second to minimize motion artefacts. Only areas which may be affected by motion artefacts are rescanned which reduces the acquisition time and thereby again motion artefacts. [6].

To sum up, the presence and severity of motion artefacts did not significantly differ among the evaluated modules and manufacturers are continuously working on better solutions to delimitate respective problems on OCT-A. For instance, some manufacturers now offer motion correction technology in order to overcome respective artefacts.

The category of imaging artefacts in our study comprised the presence of segmentation and projection artifacts. Projection artefacts arise from light, which is not directly reflected by the moving blood but passes through and illuminate features posterior to the vessel. [12] This implicates that mainly the slabs of the DCP were affected by respective artefacts in this study, while segmentation artefacts can be found in the SCP and DCP. Projection artefacts occur in all quantifying motion contrast methods irrespective whether speckle-or intensity decorrelation or phase variance is used. [18] Many manufacturers now offer software implementation which remove respective artifacts.[19] However, after removal of the projection flow signal using device inbuilt software, a “negative projection” visible as dark shadow of the same vascular pattern remains. Zeiss, Topcon as well as Optovue provided such inbuilt image processing for the removal of projection artefacts, whereas the Heidelberg prototype did not have respective post acquisition processing available at the timepoint of our evaluation. In terms of image artefacts the Zeiss and Topcon was superior to the Optovue and Heidelberg module, although just the alterations found in the SCP were statistical significant. One reason why Topcon was superior to Optovue and Heidelberg may be that SS- OCT at a wavelength of 1050 nm was used. Longer wavelengths are less susceptible to light scattering, which may decrease the presence of projection artefacts in the images of the DCP. The Angioplex uses the so-called OCT-microangiography complex (OMAG), which identifies changes in the phase and intensity information of the OCT scans. [7] The reason why the Angioplex was superior compared to the Optovue and the Heidelberg is not explainable by a longer wavelength. The use of OMAG together with the above-described advantageous implementations of the Zeiss module may account for this fact.

A recent study suggested that the usage of the FS-ADA algorithm, which is implemented in the Heidelberg module, would cause less projection artefacts compared to the SSADA algorithm.[20] The SSADA algorithm creates an isotropic voxel by degrading the axial resolution until the axial and the transverse dimension is equal, which can cause projection artefacts. In contrast, the FS-ADA algorithm detects flow from structural OCT images without impairing the axial resolution.[20] On the DCP en-face images of our analyses the Heidelberg module using FS-ADA showed indeed numerically less severe image artifacts than the Optovue, however on the SCP the Heidelberg module was inferior. This may be in line with the previous assumptions because projection artefacts are more likely to be found on the deeper en-face slabs. [12,20] Of course the inbuilt software for projection artefact removal in the Zeiss, Optovue and Topcon modules have probably also led to superior results, although in many cases “negative projections” were seen in respective cases.

But these explanations only account for the artefacts due to projections in the DCP. For the SCP mainly segmentation errors attribute to the artefacts in the category. Previous studies have shown that segmentation artefacts are rather common. [16,21] For layer segmentation different approaches are applied in each module including prepossessing steps for OCT denoising and methods such as pattern recognition, pixel classification of retinal layers, graph based multi-surface segmentation, global segmentation algorithms including active contours and Markov random fields, artificial intelligence approaches based on multiresolution hierarchic support vector machine or fuzzy C-means clustering techniques and 3D graph based methods, which lead to more or less accurate retinal layer segmentation.[22] An extensive discussion of the pros and cons of the different segmentation approaches used definitely exceeds the scope of this paper.

Interestingly the distinguishability of the FAZ borders differed between the SCP and the DCP. While the FAZ borders of the SCP was best visualized by the Zeiss Angioplex followed by the Optovue, Topcon and Heidelberg, the borders of the DCP were harder to distinguish and were best identifiable on the Optovue followed by the Heidelberg, Topcon and Zeiss module. The SSADA algorithm employed on the Optovue uses a four-fold spectrum split, which improves the signal to noise ratio, which may account for the high scoring on the distinguishability of the FAZ border on the SCP and the DCP. [7,18] It was previously shown that the SSADA algorithm provides a clean and continuous microvascular network and barely noise inside the FAZ. [7] This observation can be confirmed by our observation as the Optovue was superior in distinguishing the FAZ border in the DCP and was the second best module in differentiating the FAZ in the SCP. The ART frame on the Heidelberg device, which was set at 13 frames per B-scan, may have enhanced the signal to noise ratio and may have therefore improved the visibility of the FAZ of the DCP, which was in general harder to distinguish than the FAZ of the SCP (see scoring in Table 2). The rather inferior performance of the Topcon module in terms of respective parameter may be partly explained by the usage of a 1050nm wavelength, because spectral domain OCT using a wavelength around 800nm produces high quality angiograms with less axial scans needed and more transverse points in less time. This may be due to a lower decorrelation noise needing only two consecutive scans instead of eight for one position. [7,18] Another reaosn may be the different segmentation boundaries of each device. [23,24] Spaide et al. found that the different segmentation boundaries and their default segmentation result in different sizes and shapes of the FAZ. [24] These differences mainly originate from the inner retinal layers, which become thinner and terminate as they reach the central fovea.[24] Another paper suggested that due to the so far incorrect anatomical segmentation algorithms and the great inter-individual disparity, the best approach for the visualization of the FAZ would be the usage of the whole retina slabs. [23] Beside the above mentioned factors, the different approaches to quantify motion contrast in each module, the eye tracking and the methods to increase resolution and signal to noise ratio, other factors such as predefined contrast settings may have impacted the discrimination of the FAZ borders on each device.[25].

Vessel continuity on the SCP and DCP were best preserved and discriminable on the Zeiss module, followed by the Topcon, Optovue and Heidelberg. Of course, the severity of motion and imaging artefacts had significant impact on this evaluated parameter as high resolution and the absence of artefacts is key for the continuity and discriminability of vessels. Therefore already above mentioned parameters may account for the superior presentability of the vessels on the Zeiss module. A previous investigation indicated that the vascular network may be better visualized using OCTARA (Topcon) than the SSADA module. [8] It is further known that the SSADA algorithm mainly accounts for axial artefacts, whereas transverse artefacts, which may also cause discontinuity of vessels, remain a problem. These findings may be in line with our observations as the Topcon module was second best for the discernibility of the large vessels on the SCP and the small vessels on the DCP. However, small vessels on the SCP were better identifiable using Optovue compared to Topcon.

The vessel density of all 4 devices was evaluated using the publicly available Angiotool and was comparable among all 4 modules. The measured vessel density of the SCP in our cohort of all devices was also comparable to the normative vessel density previously reported by Coscas et al., which was evaluated with the inbuilt Angiovue software and found to be around 52.58±3.22%. [26] However although there was no difference in means, the correlation among the devices was rather weak, implicating that artefacts and differences in terms of the FAZ size and contour significantly impact the evaluation of the vessel density. This important fact should be considered when evaluating the vessel density and special attention should be drawn to the quality of the evaluated scans.

This study has definite limitations. First, a prototype was tested against three commercially available modules and it remains to be shown whether the final version of the Heidelberg porotype improves their performance compared to the other so far available modules. Second, the scanned area on the Heidelberg device (4.3x 1.5 mm) differed from the area scanned with the remaining devices (3x3mm). This may have impacted resolution and acquisition time and limits the comparability to the other devices. Usually image quality of healthy subjects outperforms quality of images of diseased eyes. Thus, the evaluation and comparison of respective modules in diseased eyes are warranted as well. Beside the 4 evaluated modules there are other OCT-A systems currently under development which were not tested in this study, including the OCT RS-3000Advance of Nidek and the SOCT Copernicus REVO and REVO NX of Optopol.

In conclusion, each device uses different approaches to offer optimal high-resolution images of the vascular network with the minimum possible artefacts. In our study, the approach of Zeiss was in most of the evaluated features superior and better than the median, but all modules had their individual strengths and weaknesses. The current study reflects just the current stage of development, but the OCT-A imaging is still in its early beginnings and is rapidly developing and improving with great strides forward.

## Supporting information

S1 Table Location of boundaries of each evaluated device outlining the superficial capillary plexus (SCP) and the deep capillary plexus (DCP). (DOCX)

S2 Table Spearman correlation coefficients among the modules in terms of vessel density. (DOCX)
