# Supplementary material for: OCT-angiography: A qualitative and quantitative comparison of 4 OCT-A devices
Source: PLoS One. 2017 May 10;12(5):e0177059. doi: 10.1371/journal.pone.0177059 (PMC5425250; doi:10.1371/journal.pone.0177059)
Supplement: S1 Table — (DOCX) [file pone.0177059.s001.docx]

S1 Table: Location of boundaries of each evaluated device outlining the superficial capillary plexus (SCP) and the deep capillary plexus (DCP)

| **Device** | **Location of boundary segmentation lines for SCP** | **Location of boundary segmentation lines for DCP** |
| --- | --- | --- |
| **Optovue** | 3 µm below ILM and 15 µm below inner boundary of IPL | 15µm below inner border of IPL and 70µm below inner border of IPL (≈ outer border of OPL) |
| **Zeiss** | ILM and outer boundary of IPL | Inner boundary of INL and outer boundary of OPL |
| **Topcon** | 2.5 µm beneath ILM and 15.6 µm beneath the interface of the IPL/INL | 15.6 µm beneath the interface of the IPL/INL and 70.2µm beneath the IPL/INL |
| **Heidelberg** | ILM and outer boundary of IPL | Outer boundary of IPL and outer boundary of OPL |

ILM= inner limiting membrane, IPL= inner plexiform layer, OPL= outer plexiform layer, INL= inner nuclear layer,
