# Supplementary material for: OCT-angiography: A qualitative and quantitative comparison of 4 OCT-A devices
Source: PLoS One. 2017 May 10;12(5):e0177059. doi: 10.1371/journal.pone.0177059 (PMC5425250; doi:10.1371/journal.pone.0177059)
Supplement: S2 Table — (DOCX) [file pone.0177059.s002.docx]

S2 Table: Spearman correlation coefficients among the modules in terms of vessel density

| **Device** | **Optovue** | **Zeiss** | **Topcon** | **Heidelberg** |
| --- | --- | --- | --- | --- |
| **Optovue** | 1 | 0.14 | 0.1 | -0.16 |
| **Zeiss** | 0.14 | 1 | 0.2 | -0.06 |
| **Topcon** | -0.16 | 0.2 | 1 | 0.35 |
| **Heidelberg** | 0.14 | -0.06 | 0.35 | 1 |
